# Exogenously Applied Gibberellic Acid Enhances Growth and Salinity Stress Tolerance of Maize through Modulating the Morpho-Physiological, Biochemical and Molecular Attributes

**DOI:** 10.3390/biom11071005

**Published:** 2021-07-09

**Authors:** Kashif Shahzad, Sadam Hussain, Muhammad Arfan, Saddam Hussain, Ejaz Ahmad Waraich, Shahid Zamir, Maham Saddique, Abdur Rauf, Khaled Y. Kamal, Christophe Hano, Mohamed A. El-Esawi

**Affiliations:** 1 Department of Botany, University of Agriculture Faisalabad, Faisalabad 38040, Pakistan; kashifshahzad623@gmail.com (K.S.); mi_anwar@yahoo.com (M.A.); mahumsaddique@yahoo.com (M.S.); 2 Department of Agronomy, University of Agriculture Faisalabad, Faisalabad 38040, Pakistan; ch.sadam423@gmail.com (S.H.); ewaraich@uaf.edu.pk (E.A.W.); zamir757@gmail.com (S.Z.); 3 College of Agronomy, Northwest A&F University, Yangling 712100, China; 4 Department of Chemistry, University of Swabi, Anbar 23430, Pakistan; mashaljcs@yahoo.com; 5 Agronomy Department, Faculty of Agriculture, Zagazig University, Zagazig 44519, Egypt; kymoustafa@tamu.edu; 6 Laboratoire de Biologie des Ligneux et des Grandes Cultures (LBLGC), INRAE USC1328, Université d’Orléans, 28000 Chartres, France; hano@univ-orleans.fr; 7 Botany Department, Faculty of Science, Tanta University, Tanta 31527, Egypt

**Keywords:** salinity, antioxidants, gibberellic acid, seed priming, ionic balance, gene expression

## Abstract

Soil salinity is the major limiting factor restricting plant growth and development. Little is known about the comparative and combined effects of gibberellic acid (GA_3_) seed priming and foliar application on maize under salt stress. The current study determined the impact of different application methods of GA_3_ on morpho-physiological, biochemical and molecular responses of maize seedlings under three salinity stress treatments (no salinity, moderate salinity-6 dS m^−1^, and severe salinity-12 dS m^−1^). The GA_3_ treatments consisted of control, hydro-priming (HP), water foliar spray (WFS), HP + WFS, seed priming with GA_3_ (GA_3_P, 100 mg L^−1^), foliar spray with GA_3_ (GA_3_FS, 100ppm) and GA_3_P + GA_3_FS. Salt stress particularly at 12 dS m^−1^ reduced the length of shoots and roots, fresh and dry weights, chlorophyll, and carotenoid contents, K^+^ ion accumulation and activities of antioxidant enzymes, while enhanced the oxidative damage and accumulation of the Na^+^ ion in maize plants. Nevertheless, the application of GA_3_ improved maize growth, reduced oxidative stress, and increased the antioxidant enzymes activities, antioxidant genes expression, and K^+^ ion concentration under salt stress. Compared with control, the GA_3_P + GA_3_FS recorded the highest increase in roots and shoots length (19–37%), roots fresh and dry weights (31–43%), shoots fresh and dry weights (31–47%), chlorophyll content (21–70%), antioxidant enzymes activities (73.03–150.74%), total soluble protein (13.05%), K^+^ concentration (13–23%) and antioxidants genes expression levels under different salinity levels. This treatment also reduced the H_2_O_2_ content, and Na^+^ ion concentration. These results indicated that GA_3_P + GA_3_FS could be used as an effective tool for improving the maize growth and development, and reducing the oxidative stress in salt-contaminated soils.

## 1. Introduction

Maize (*Zea mays* L.) is a staple cereal crop, cultivated throughout the world for its usage as forage, and food grains for human and animal feed. It also provides raw materials to various industries [1]. Maize crop is subjected to various abiotic stresses under field conditions, such as soil salinity, drought, light and temperature, which may lead to a severe decline in its productivity [2]. Among abiotic stresses, soil salinity is one of the major factors limiting crop growth and productivity. It is estimated that about 6% of the total arable land worldwide is affected by soil salinity [3]. Soil salinity not only decreases the seed emergence and germination rates but also severely reduces the growth, development and yield of field crops [4,5]. Also, high salt concentrations in the soil lead to the closure of stomata, and damage photosynthetic machinery and chlorophyll content [6,7,8]. In plants, reactive oxygen species (ROS) are continuously produced as a result of metabolic activities, with more production under environmental stress stimuli [4]. The increase in ROS production can lead to the destruction of lipids, membranes, nucleic acids and proteins, and results in malfunction of cellular machinery [9,10,11]. The overproduction of ROS under salt stress also imposes a disturbance in ion balance [12,13]. In order to reduce the oxidative damage, plants have developed an effective antioxidant defense system comprising of antioxidant enzymes like superoxide dismutase (SOD), peroxidase (POD), ascorbate peroxidase (APX), catalase (CAT) [14].

Gibberellic acid (GA_3_) application can help to improve plant growth and development under salt stress as it improves the pigment content [15] and reduces the Na^+^ concentration in shoots and roots [16]. The application of GA_3_ through seed priming reduces the emergence time by increasing the water absorption and metabolic activities in seeds [17]. When applied at low concentrations, GA_3_ uplifts the seed dormancy, increases plant growth and overall plant productivity [18]. Gibberellic acid increases the growth of root, shoot and number of leaves by altering the process of cell division and cell elongation [19]. In maize, GA_3_ application caused a remarkable increase in total chlorophyll contents under salinity [20]. Under salt stress, GA_3_ application enhanced the dry matter production and plant growth of wheat because of increased photosynthetic activities [21]. In another study, seed priming with GA_3_ significantly improved the plant height, yield and yield-related traits, Ca^2+^ and K^+^ concentrations, and transpiration rates while decreased Na^+^ concentrations in wheat under salt stress [15]. Although, several studies in recent years have discussed the beneficial role of GA_3_ application (either as seed priming or foliar spray) for the different field crops including maize [20] wheat [21,22] and rice [23], yet little is known regarding the comparative and combined effects of GA_3_-seed priming and GA_3_-foliar spray on morpho-physiological, biochemical and molecular responses of maize seedlings under salinity stress. It was hypothesized that GA_3_, when applied as combine foliar and seed priming, may effectively alleviate the salinity stress in maize by reducing oxidative stress damage and increasing plant growth and biomass production. The specific objectives of the present study were (a) to investigate the impact of GA_3_ as a foliar spray and/or seed priming on the growth, physiological parameters, and ionic homeostasis in maize under salinity stress, and (b) to underpin the mechanism of GA_3_-induced salt stress tolerance in maize.

## 2. Materials and Methods

### 2.1. Plant Material and Experimental Site

The seeds of maize cultivar (Hybrid-30T60) were obtained from the Maize Research Institute, Ayub Agricultural Research Institute, Faisalabad, Pakistan. Experimental soil used in pots contained 2.54 g kg^−1^ soil organic carbon, 45.05 mmol L^−1^ total soluble salts, 30.89 mmol L^−1^ sodium (Na^+^), 0.45 mmol L^−1^ potassium (K^+^) and 20.88 mmol L^−1^ chloride (Cl^-^) content. The experiment was carried out in the greenhouse at the Old Botanical Garden University of Agriculture Faisalabad, Pakistan (longitude = 73°05′ E, latitude = 31°44′ N and 184.4 m above sea level) during winter season (December 2018–January 2019).

### 2.2. Experimentation

The pots, having 25 cm depth and 15.25 cm diameter, were filled with 7 kg of well sieved soil. Fertilizers were thoroughly mixed in the soil at the equivalent of 2.50 g N pot^−1^, 1.4 P_2_O_5_ pot^−1^ and 0.9 g K_2_O pot^−1^ (250: 140: 90 kg NPK hectare^−1^, respectively). Maize seeds (hybrid-30T60) were disinfected and sown on 12 December 2018, and seeds in each pot were equally spaced. The irrigation was applied on daily basis to maintain the field capacity of the soil at 80%. The weeds from each pot were eliminated manually.

### 2.3. Treatments and Experimental Design

This experiment consisted of two factors: salinity and gibberellic acid (GA_3_) application. Salt stress was imposed by sodium chloride, and three treatments were as follows: (1) No salinity; (2) moderate salinity (6 dS m^−1^); (3) and severe salinity (12 dS m^−1^). A procedure of USDA Laboratory staff (1954) was followed to maintain the required salt level. The salinity was imposed prior to sowing during the filling the pots. There were seven treatments for GA_3_ application which included a control; hydro-priming (HP); water foliar spray (WFS); HP + WFS; seed priming with GA_3_ (GA_3_P, 100 mg L^−1^); foliar spray with GA_3_ (GA_3_FS, 100 ppm); and GA_3_P + GA_3_FS. For seed priming, maize seeds were soaked at room temperature in distilled water (for hydro-priming) and in GA_3_ solution (100 mg L^−1^), using seed weight to solution volume ratio of 1:5. After 12 h, seeds were thoroughly washed with distilled water thrice and re-dried to their original weight. Treatments of WFS (distilled water) and GA_3_FS (100 ppm) were imposed at 15 DAS in equal volume (50 mL/pot). In total, there were 21 treatments with three replications, and the treatments were arranged in a completely randomized design (CRD). Ten days after the foliar application of gibberellic acid, the plants were harvested for measuring the observed traits.

### 2.4. Measurement of Plant Growth Attributes

At 25 DAS, the plants were harvested to measure the root and shoot length, fresh and dry weights, leaf length, and leaf width. A calibrated meter rod was used to measure the seedling length. After measuring the fresh weight, the roots and shoots were dried in the sun for 96 h, and then kept in an electric oven at 70 °C until constant weight, and the dry weight was calculated with an electric balance.

### 2.5. Determination of Photosynthetic Pigments

The chlorophyll (chlorophyll a (Chla), chlorophyll b (Chlb), Chla + Chlb, and carotenoid contents were determined by the method suggested by Arnon [24] with some modifications. The 0.1 g of fresh and healthy leaves were crushed, and extraction was done with 5 mL of acetone (80%). The extract was transferred to a test tube and left in the dark for 24 h. The absorbance of the supernatant was recorded at wavelengths 663, 645, and 480 nm by using a spectrophotometer. The chlorophyll and carotenoid contents were computed by following these equations and expressed in mg g^−1^ fresh weight:

Chla = [(0.0127 × A663 − 0.00269 × A645) × 100]/0.5

Chlb = [(0.0229 × A645 − 0.00468 × A663) × 100]/0.5

Carotenoids = Acar/Em × 100

where, A663 and A645 are the corresponding wavelengths of light density values, Acar = OD 480 + 0.114 (OD663) − 0.638 (OD645) and Em = 2500.

### 2.6. Determination of Hydrogen Peroxide (H_2_O_2_)

The H_2_O_2_ content was evaluated spectrophotometrically [14]. In a pre-chilled mortar and pestle, about 0.5 g of fresh leaf tissues were ground in 0.1% Trichloroacetic acid (TCA). The extract was then centrifuged at 12,000 rpm for 15 min. The reaction sample composed of 0.5 mL of supernatant, 1 mL of 1M potassium iodide (KI), and 0.5 mL of potassium phosphate buffer was used, and the absorbance was recorded at 390 nm wavelength.

### 2.7. Determination of Antioxidant Enzymes Activities

Fresh leave tissues (0.25 g) were grounded in 5 mL of potassium phosphate buffer (pH 7.8) with a mortar and pestle and then centrifuged at 15,000 rpm. The activity of catalase (CAT) and peroxidase (POD) enzymes was measured by the method of Chance [25]. For the CAT enzyme, a reaction mixture comprised of 1.9 mL of phosphate buffer (50 mM), 100 µL of extract sample, and 100 µL of H_2_O_2_ was prepared, and the absorbance was recorded on a spectrophotometer at a wavelength of 240 nm. For POD activity, a reaction mixture comprised of 750 µL of phosphate buffer (50 mM), 100 µL of H_2_O_2_, 10 µL of guaiacol, and 50 µL sample was used, and the absorbance was recorded at 470 nm. The activity of superoxide dismutase was measured spectrophotometrically [26] using the Nitro blue tetrazolium (NBT). The reaction mixture of 250 µL of phosphate buffer (7.8 pH), 100 µL of triton, 50 µL of NBT, 100 µL of L-methionine, 400 µL of distilled water, 50 µL of riboflavin, and 50 µL of the extract was prepared, and the absorbance was recorded at 560 nm.

### 2.8. Analysis of Antioxidant Genes Expression

The antioxidant gene expression levels (i.e., *SOD*, *POD*, and *CAT*) were evaluated in maize plants by quantitative real-time PCR analysis. Total RNA and cDNA were extracted and synthesized from plant tissues using the RNeasy Plant Mini kit and Reverse Transcription kit (Qiagen, Germany), respectively. Using the protocol of QuantiTect SYBR Green PCR kit (Qiagen, Germany), PCR reactions were performed in triplicates. Amplification procedures were done as follows: 95 °C for 10 min; 40 cycles of 95 °C for 20 s, 60 °C for 30 s, 72 °C for 2 min, 72 °C for 4 min. A melting-curve analysis was assayed to check the amplification specificity. *SOD*, *POD* and *CAT* genes primers [27,28] were used for amplification. *Actin2* was assayed as a reference gene [28] and the relative gene expression levels were estimated using the 2^−ΔΔCt^ method.

### 2.9. Total Soluble Proteins Determination

The total soluble protein was determined by using the method of Bradford [29]. About 0.5 g of fresh leaves were grounded in 10 mL of 50 mM potassium phosphate buffer [K_2_HPO_4_ + KH_2_PO_4_] in the ice bath. The extract was then centrifuged at 10,000 rpm for 15 min at 4 °C. The absorbance mixture containing 100 mL of H_2_PO_4_, 5 μL of extract sample, 95 μL of NaCl, and 1 mL of Bradford dye was prepared, and the absorbance was recorded at 595 nm with a spectrophotometer.

### 2.10. Estimation of Phenolic Contents

In this study, the total phenolic contents were determined according to Ainsworth and Gillespie [30]. About 0.5 g leaves were extracted in 5 mL of 80% acetone, and after filtration, the volume was increased to 10 mL by using acetone. The reaction mixture comprised 20 μL of sample solution, 1.58 mL of water, and 100 μL of Folin-Ciocalteu reagents. The reaction sample was then mixed with 300 μL of sodium carbonate and kept at 40 °C for 30 min. The absorbance of the reaction sample was recorded at 760 nm using a spectrophotometer and the phenolic content was computed using the standard curve stated by Ainsworth and Gillespie [30].

### 2.11. Mineral Ions Determination (Na^+^, K^+^ and Ca^2+^) in Plant Tissues

In this study, the Flame photometer (flame photometer 410) was used to determine the mineral ions (Na^+^, K^+^, and Ca^2+^) in plant tissues [31]. Digest about 0.1 g dried sample in 2 mL H_2_SO_4_ in the digestion flask, and then placed the sample at room temperature for overnight. The samples were then heated at 150 °C by using the hot plate. The samples were grinded in 2 mL of H_2_O_2_ and use distilled water to increase the volume to 50 mL. Then, sodium (Na^+^), potassium (K^+^) and calcium (Ca^2+^) ions were determined by using a Flame photometer (Flame photometer 410).

### 2.12. Statistical Analysis

The growth, physiological and biochemical observations were proceeded to Microsoft Excel for the calculation of mean values and standard deviation. The ANOVA was used to statistically analyze the data, and multiple comparisons were performed using the least significant difference test at *p*-value of less than 5% (*p* < 0.05) to compare the treatment means [32] using Statistix 8.1. SigmaPlot was used for graphical presentation.

## 3. Results

### 3.1. Fresh and Dry Weight of Roots and Shoots

**Figure 1:**
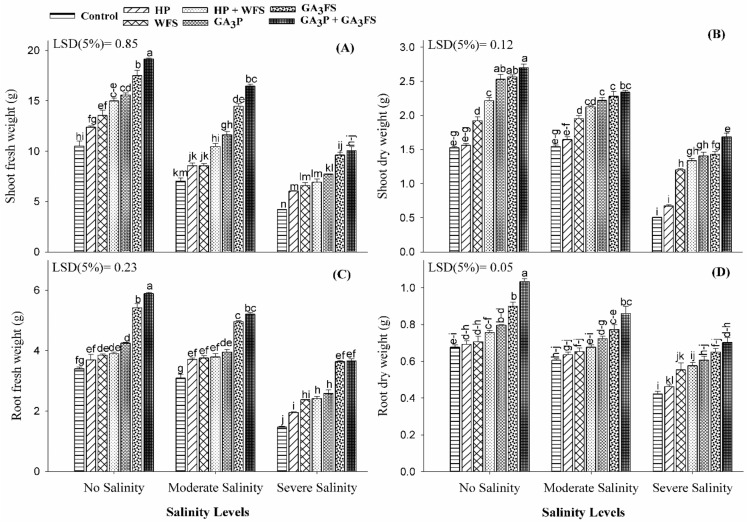
Shoot fresh weight (**A**), shoot dry weight (**B**), root fresh weight (**C**) and root dry weight (**D**) of maize as affected by different treatments of gibberellic acid under no salinity (control), moderate salinity (6 dS m^−1^), and severe salinity (12 dS m^−1^). Values are means ± SD (n = 3). Hydropriming (HP); water foliar spray (WFS); hydropriming and water foliar spray (HP + WFS); seed priming with GA_3_ (GA_3_P); foliar spray with GA_3_ (GA_3_FS); seed priming and foliar spray of GA_3_ (GA_3_P + GA_3_FS). Bars with the same letters do not differ significantly at *p* ≤ 0.05.

Plant growth significantly differed with gibberellic acid (GA_3_) application under salinity stress (Figure 1; Table S1). Salt stress significantly reduced the fresh and dry weight of roots and shoots as compared to no salinity treatment control (Figure 1). As compared to the control, moderate and severe salinity treatments reduced the shoot fresh weight by 34.27% and 102.59%, the shoot dry weight by 6.48% and 82.07%, the root fresh weight by 6.76% and 68% and root dry biomass by 12.84% and 39.93%, respectively. The application of GA_3_ significantly increased the fresh and dry weight of roots and shoots than control (Figure 1). Under severe salinity, GA_3_ priming with foliar spray (GA_3_P + GA_3_FS) increased the shoot fresh and dry weight, root fresh weight and dry weight by 138.71%, 232.89%, 145.08% and 67.19%.

### 3.2. Seedling Length and Leaves Growth

**Figure 2:**
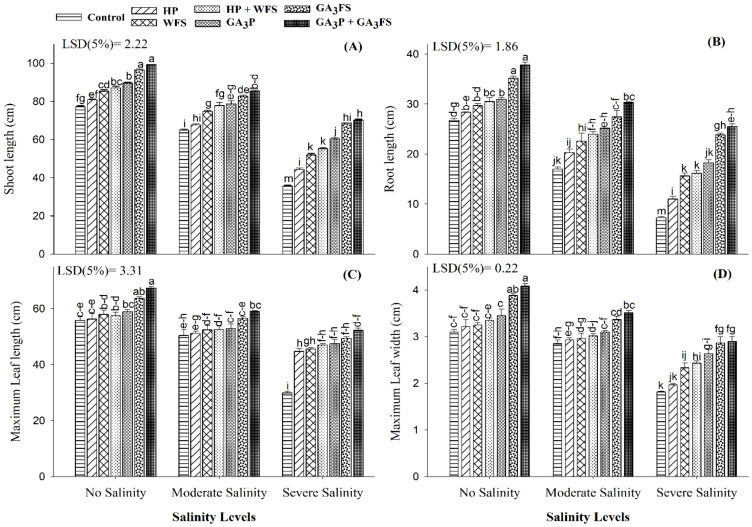
Shoot length (**A**), root length (**B**), maximum leaf length (**C**) and maximum leaf width (**D**) of maize as affected by different treatments of gibberellic acid under no salinity (control), moderate salinity (6 dS m^−1^), and severe salinity (12 dS m^−1^). Values are means ± SD (n = 3). Hydropriming (HP); water foliar spray (WFS); hydropriming and water foliar spray (HP + WFS); seed priming with GA_3_ (GA_3_P); foliar spray with GA_3_ (GA_3_FS); seed priming and foliar spray of GA_3_ (GA_3_P + GA_3_FS). Bars with the same letters do not differ significantly at *p* ≤ 0.05.

When plants were exposed to salinity stress, shoot and root length, maximum leaf length and width were reduced significantly, but the application of GA_3_ alleviated the damage caused by salinity stress (Figure 2). Plants treated with GA_3_ had a higher shoot and root length, and maximum leaf length and width compared to the control; the maximum values were reported for GA_3_P + GA_3_FS treatment. Under severe salinity, GA_3_P + GA_3_FS application increased the shoot and root length, maximum leaf length and width by 96.86%, 251.95%, 74.65% and 59.63%, respectively.

### 3.3. Chlorophyll and Carotenoid Content

**Figure 3:**
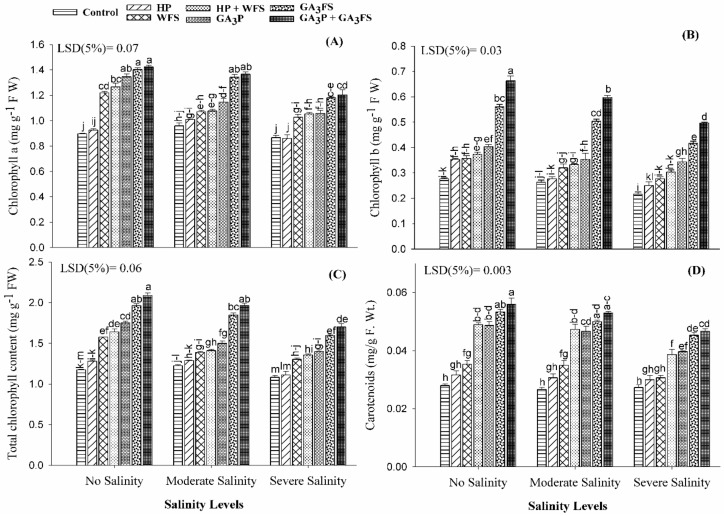
Chlorophyll a (**A**), chlorophyll b (**B**), total chlorophyll (**C**) and carotenoid content (**D**) of maize as affected by different treatments of gibberellic acid under no salinity, moderate salinity (6 dS m^−1^), and severe salinity (12 dS m^−1^). Values are means ± SD (n = 3). Hydropriming (HP); water foliar spray (WFS); hydropriming and water foliar spray (HP + WFS); seed priming with GA_3_ (GA_3_P); foliar spray with GA_3_ (GA_3_FS); seed priming and foliar spray of GA_3_ (GA_3_P + GA_3_FS). Bars with the same letters do not differ significantly at *p* ≤ 0.05.

Salinity stress significantly reduced the Chla, Chlb and total chlorophyll content in maize leaves (Figure 3A–C; Table S1). Under severe salinity, Chla, Chlb and total chlorophyll were decreased by 17.06%, 29.66% and 20.10% respectively compared with no salinity treatment. The application of GA_3_ significantly increased the chlorophyll content compared with the corresponding treatments without GA_3_ application; the maximum increase in chlorophyll content was recorded by GA_3_P + GA_3_FS. Under severe salinity, GA_3_P + GA_3_FS increased the Chla, Chlb and total chlorophyll by 58.73%, 139.75% and 77.84%, respectively compared to control plants. Overall, the positive influence of GA_3_ treatments for chlorophyll content was in the order of GA_3_P + GA_3_FS > GA_3_FS > GA_3_P > HP + WFS > WFS > HP. Interestingly, foliar spray of water also significantly improved the chlorophyll content as compared with hydropriming and control plants, even under non-stressed conditions.

Salt stress decreased the carotenoid content than the control (Figure 3D). Under severe salinity, carotenoid content was 16.90% lower than the non-stressed plants. The GA_3_ application significantly increased the carotenoid content than respective treatments without GA_3_ application. Under severe salinity, GA_3_FS and GA_3_P + GA_3_FS increased the carotenoid content by 65.86% and 70.73% respectively compared with the control.

### 3.4. Hydrogen Peroxide (H_2_O_2_) Concentration

**Figure 4:**
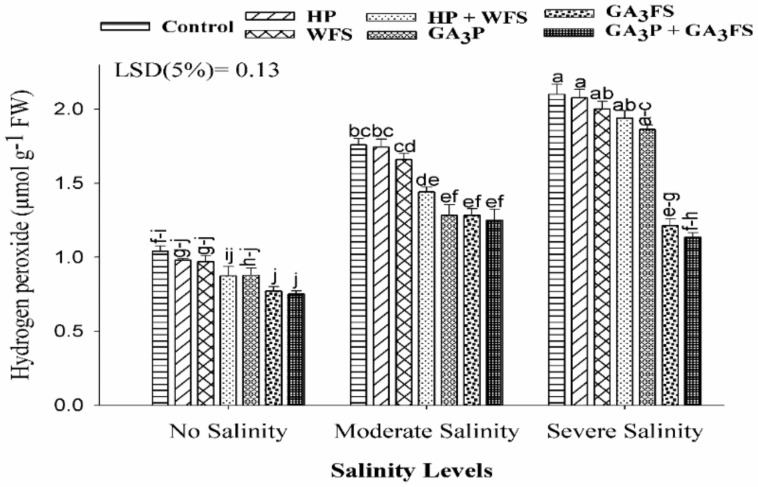
Hydrogen peroxide (H_2_O_2_) content in maize leaves as affected by different treatments of gibberellic acid under no salinity, moderate salinity (6 dS m^−1^), and severe salinity (12 dS m^−1^). Values are means ± SD (n = 3). Hydropriming (HP); water foliar spray (WFS); hydropriming and water foliar spray (HP + WFS); seed priming with GA_3_ (GA_3_P); foliar spray with GA_3_ (GA_3_FS); seed priming and foliar spray of GA_3_ (GA_3_P + GA_3_FS). Bars with the same letters do not differ significantly at *p* ≤ 0.05.

Salinity stress enhanced the H_2_O_2_ concentration in maize leaves (Figure 4). The highest concentration was observed in severe salinity without GA_3_ application which was 12% higher than non-stressed plants. The GA_3_ application significantly (*p* < 0.05) reduced the H_2_O_2_ content than respective hydro-treatments and control. Under severe salinity, the maximum reduction was reported with GA_3_P + GA_3_FS treatment, which decreased the H_2_O_2_ content by 85.84% than control.

### 3.5. Antioxidant Activities

**Figure 5:**
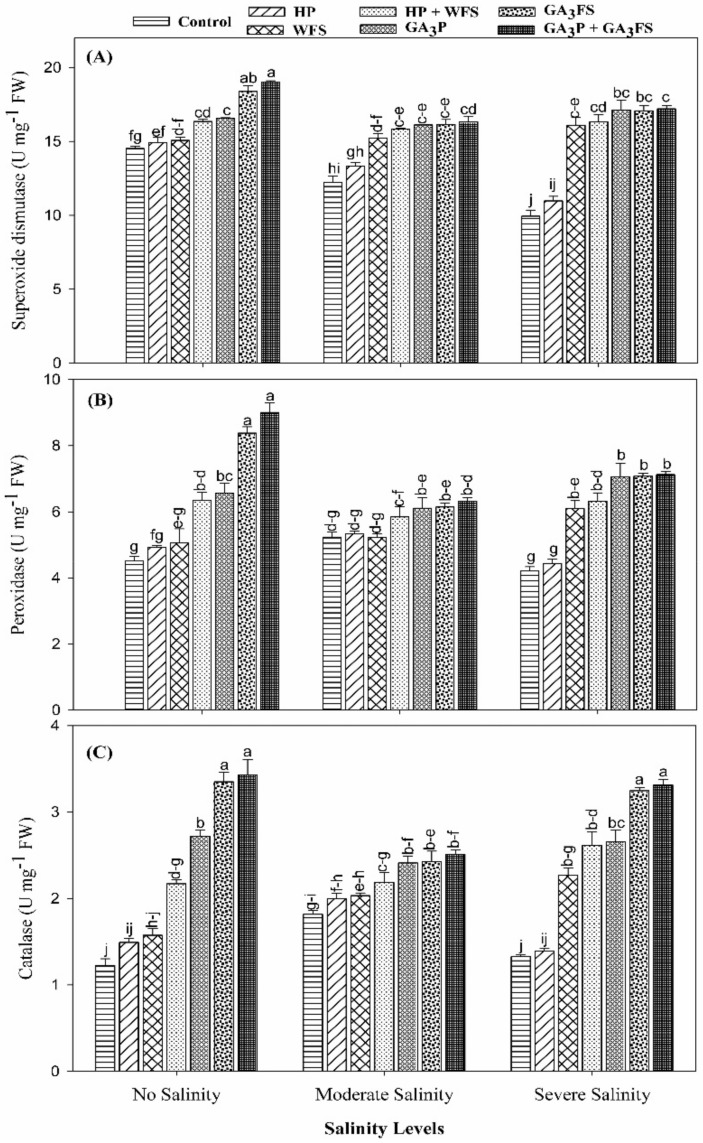
Superoxide dismutase (**A**), peroxidase (**B**) and catalase (**C**) activity in maize leaves as affected by different treatments of gibberellic acid under no salinity (control), moderate salinity (6 dS m^−1^), and severe salinity (12 dS m^−1^). Values are means ± SD (n = 3). Hydropriming (HP); water foliar spray (WFS); hydropriming and water foliar spray (HP + WFS); seed priming with GA_3_ (GA_3_P); foliar spray with GA_3_ (GA_3_FS); seed priming and foliar spray of GA_3_ (GA_3_P + GA_3_FS). Bars with the same letters do not differ significantly at *p* ≤ 0.05.

The application of GA_3_ and salt stress significantly affected the activities of SOD, POD, and CAT in maize leaves (Figure 5; Table S1).

Salinity stress caused a marked reduction in the activities of SOD, POD and CAT compared with the unstressed treatment. Nonetheless, GA_3_ application significantly (*p* < 0.05) increased the activities of antioxidant enzymes under salinity as well as the unstressed conditions. At moderate salinity, GA_3_ application increased the activities of SOD, POD and CAT in the range of 12.23–38.12% relative to the control plants. Under severe salinity, the maximum increase in SOD, POD and CAT enzyme activities was reported for GA_3_P + GA_3_FS, which were 73.03%, 69.52% and 150.74% than respective control, however, these values were statistically similar with GA_3_FS treatments.

### 3.6. Expression Analysis of Antioxidant Genes

**Figure 6:**
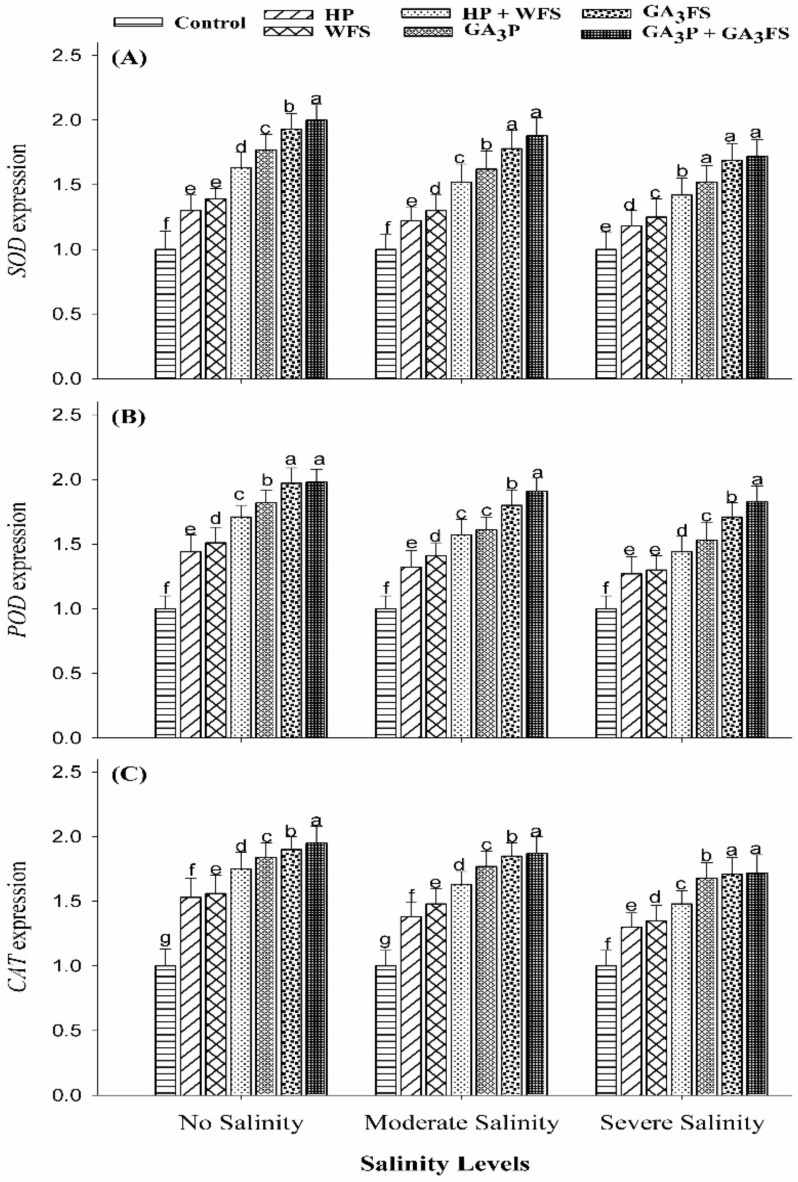
Expression level of *SOD* gene (**A**), *POD* gene (**B**) and *CAT* gene (**C**) in maize leaves as affected by different treatments of gibberellic acid under no salinity (control), moderate salinity (6 dS m^−1^), and severe salinity (12 dS m^−1^). Values are means ± SD (n = 3). Hydropriming (HP); water foliar spray (WFS); hydropriming and water foliar spray (HP + WFS); seed priming with GA_3_ (GA_3_P); foliar spray with GA_3_ (GA_3_FS); seed priming and foliar spray of GA_3_ (GA_3_P + GA_3_FS). Bars with the same letters do not differ significantly at *p* ≤ 0.05.

The application of GA_3_ and salt stress significantly affected the expression of antioxidant genes (*SOD, POD,* and *CAT*) in maize leaves (Figure 6).

Severe salinity stress caused a marked decrease in the expression levels of antioxidant genes (*SOD, POD,* and *CAT*) compared with unstressed plants (Figure 6). Nonetheless, GA_3_ application significantly (*p* < 0.05) enhanced the expression levels of antioxidant genes particularly under moderate and severe salinity. Under severe salinity, the maximum increase in the expression levels of antioxidant genes (*SOD, POD,* and *CAT*) was recorded for GA_3_P + GA_3_FS (Figure 6).

### 3.7. Total Soluble Protein and Total Phenolics

**Figure 7:**
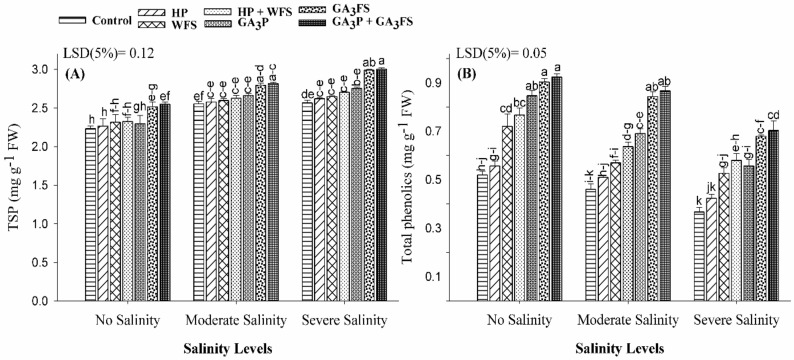
Total soluble protein-TSP (**A**) and total phenolic content (**B**) of maize as affected by different treatments of gibberellic acid under no salinity (control), moderate salinity (6 dS m^−1^), and severe salinity (12 dS m^−1^). Values are means ± SD (n = 3). Hydropriming (HP); water foliar spray (WFS); hydropriming and water foliar spray (HP + WFS); seed priming with GA_3_ (GA_3_P); foliar spray with GA_3_ (GA_3_FS); seed priming and foliar spray of GA_3_ (GA_3_P + GA_3_FS). Bars with the same letters do not differ significantly at *p* ≤ 0.05.

Salinity stress significantly affected the total soluble protein contents in maize leaves as compared with unstressed plants (Figure 7A). The total soluble protein was increased significantly under GA_3_ application, the maximum values were recorded for GA_3_P + GA_3_FS indicating that the application of GA_3_ as seed priming and foliar spray effectively enhance the soluble protein under salinity stress. Salinity stress significantly reduced the total phenolic content in maize leaves, and the maximum reduction was found under severe salinity (Figure 7B). Under severe salinity, GA_3_P + GA_3_FS increased the phenolic content by 70.72% compared with the respective control. Averaged across different stress treatment, the increase in phenolics contents was in the order of GA_3_P + GA_3_FS > GA_3_FS > GA_3_P > HP + WFS > WFS > HP. Interestingly, foliar water spray also significantly increased the total phenolic content as compared to control plants, even under non-stressed conditions.

### 3.8. Mineral Ions (Na^+^, K^+^ and Ca^2+^) Concentrations

**Figure 8:**
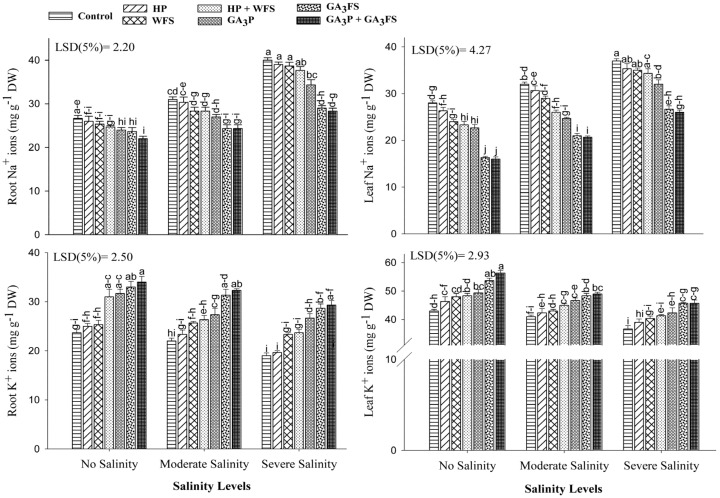
Sodium (Na^+^) and potassium (K^+^) ion concentration in different maize parts as affected by different treatments of gibberellic acid under no salinity (control), moderate salinity (6 dS m^−1^), and severe salinity (12 dS m^−1^). Values are means ± SD (n = 3). Hydropriming (HP); water foliar spray (WFS); hydropriming and water foliar spray (HP + WFS); seed priming with GA_3_ (GA_3_P); foliar spray with GA_3_ (GA_3_FS); seed priming and foliar spray of GA_3_ (GA_3_P + GA_3_FS). Bars with the same letters do not differ significantly at *p* ≤ 0.05.

Salinity stress and GA_3_ application exhibited a significant effect on Na^+^ and K^+^ ion concentrations in different parts of maize plants (Figure 8; Table S1). Salt stress significantly increased the Na^+^ ion concentration in the roots and leaves as compared to unstressed plants. Severe salinity caused the highest increase in Na^+^ ions in roots and leaves, which was about 30.22% and 47.43%, higher than unstressed treatment, respectively. The application of GA_3_ significantly reduced the Na^+^ ion concentrations in roots and leaves than the control. Under severe salinity, GA_3_P, GA_3_FS and GA_3_P + GA_3_FS lowered the Na^+^ ion concentration in roots by 16.50%, 37.93% and 41.17%, respectively compared to the control. Similarly, GA_3_ significantly reduced the Na^+^ ion concentration in leaves, with maximum reduction under GA_3_P + GA_3_FS, which was 31.56% lower than control under severe salinity stress. Salinity stress reduced the accumulation of K^+^ ion in different plant parts; the lowest concentration of K^+^ ion in roots and shoots was found under severe salinity. The application of GA_3_ significantly increased the concentration of K^+^ ions in shoots and roots, with the maximum increase in GA_3_P + GA_3_FS compared with control. Under severe salinity stress, GA_3_P + GA_3_FS increased the concentration of K^+^ ions in roots and shoots by 54.38% and 20.13%, respectively than the respective control.

**Figure 9:**
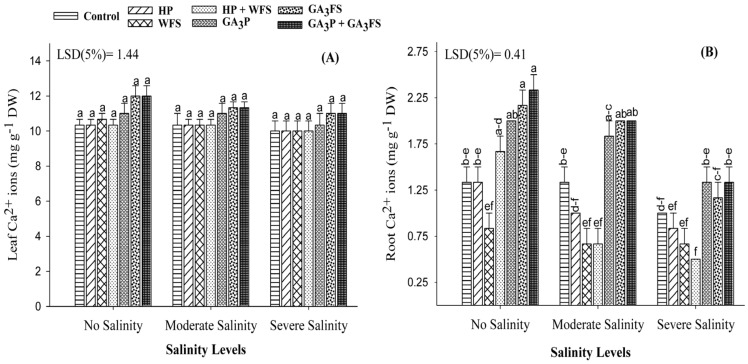
Calcium (Ca^2+^) ions in maize shoots (**A**) and roots (**B**) as affected by different treatments of gibberellic acid under no salinity (control), moderate salinity (6 dS m^−1^), and severe salinity (12 dS m^−1^). Values are means ± SD (n = 3). Hydropriming (HP); water foliar spray (WFS); hydropriming and water foliar spray (HP + WFS); seed priming with GA_3_ (GA_3_P); foliar spray with GA_3_ (GA_3_FS); seed priming and foliar spray of GA_3_ (GA_3_P + GA_3_FS). Bars with the same letters do not differ significantly at *p* ≤ 0.05.

In this study, a non-significant difference (*p* > 0.05) for Ca^2+^ ion in maize shoot was observed among salt stress and GA_3_ treatments (Figure 9A; Table S1). However, salinity and GA_3_ treatments significantly influenced the Ca^2+^ ions in maize roots. Salinity stress significantly decreased the accumulation of Ca^2+^ ions in maize roots compared to the unstressed treatment, and the maximum reduction was recorded under severe salinity (Figure 9B). The GA_3_-treated plants had higher concentrations of Ca^2+^ ion in roots as compared with non-GA treated plants. Under moderate and severe salinity, GA_3_P + GA_3_FS increased the concentration of Ca^2+^ ions in maize roots by 59.25% and 28.57% than their respective control (Figure 9B).

## 4. Discussion

Abiotic stresses restrict plant growth and development [33,34]. The present study demonstrated that salinity stress, particularly at 12 dS m^−1^, significantly hampered the plant growth and biomass accumulation compared the unstressed control (Figure 1 and Figure 2). Consistently, several previous studies [35,36] have also documented that salinity is highly detrimental to plant growth and biomass production. Salt stress in maize reduced the root and shoot length, dry and fresh biomass and leaves growth compared to the control [36]. The decrease in plant biomass might be due to the higher Na^+^ ion concentration in roots and outside the plant cell [35,37]. In the soil, higher salt concentration limits the absorption of water and nutrients by plant roots. Moreover, the higher accumulation of Na^+^ ions in roots can cause osmotic stress, reduce water potential, and disturb the nutrient balance in plants. The higher concentrations of Na^+^ inside and outside the plant cell have a negative impact on K^+^ influx in the cell, the latter being an essential element required for plant growth [35,37]. In the present study, application of GA_3_ as priming and/or foliar spray significantly improved the growth of maize crop under salinity stress (Figure 1 and Figure 2). The beneficial effect of GA_3_-seed priming or GA_3_-foliar spray has previously been reported by several researchers in different field crops [17,19,20,21,22,38]. The exogenous supply of GA_3_ may enhance its endogenous accumulation, which may facilitate the better growth of plants [15]. The GA_3_ is considered as an important hormone for cell elongation, therefore, the better growth and seedling length of GA_3_-treated maize in the present study (Figure 1 and Figure 2) might be due to the higher cell and stem elongation [20,39]. The application of GA_3_ can also increase the plant growth because of improved carbohydrate metabolism [40]. In the past, GA_3_ application-induced growth stimulation of wheat under zinc oxide nanoparticle stress was attributed to improved nutritional status [22].

Chlorophyll is the main pigment of plant photosynthesis and plays an important role in different physiological processes of plants [41]. In this study, salinity stress significantly reduced leaf chlorophyll content (shown as Chla, Chlb and total chlorophyll) as compared to untreated plants (Figure 3). Various previous reports have also confirmed that salt stress can reduce the activity of photosynthetic pigments [42,43]. The decrease in chlorophyll content can be attributed to the formation of proteolytic enzymes at high salt concentrations [20]; and these enzymes are responsible for the degradation of chlorophyll [44], and reduction of photosynthesis under salt conditions [45]. The reduction in maize biomass under salt stress (Figure 1 and Figure 2) may also be due to the decrease in chlorophyll contents (Figure 3) and photosynthesis rate [46]. The application of GA_3_ increased the chlorophyll content of maize leaves exposed to salt stress, with a maximum increase in case of the GA_3_P + GA_3_FS treatment (Figure 3). The higher accumulation of chlorophyll content in GA_3_-treated maize seedlings under salinity stress might be linked to lower Na^+^ accumulation (Figure 8), lower oxidative damage and an improved antioxidant defense system (Figure 4, Figure 5 and Figure 6). These findings are consistent with the results of previous studies [47,48,49], which reported that the application of GA_3_ increased leaf chlorophyll content. Foliar applied GA_3_ significantly improved the chlorophyll content in maize under salt stress [20]. In our work, seed priming and foliar application of GA_3_ decreased Na^+^ contents in maize tissue, suggesting that translocation of Na^+^ from roots to shoots might be inhibited by GA_3_ treatments (Figure 8). Less accumulation of Na^+^ in maize tissue might show less harmful effects on leaf functions, which may affect the changes of photosynthetic pigments in the present study (Figure 3). Additionally, in our work, the positive influence of GA_3_ treatments was more pronounced for chlorophyll b (Figure 3). However, the mechanism behind this requires further exploration.

Oxidative stress has an adverse effect on plant cell functions [50]. Under oxidative stress, excessive production of ROS leads to damage to plant tissue [51]. More production of ROS under salt stress has been reported before [52,53,54]. Consistent with published studies, higher concentrations of H_2_O_2_ under salt stress were observed in maize seedlings (Figure 4). However, plants have evolved many defense mechanisms to reduce ROS-induced damage by modulating the activities of the antioxidant enzymes as well as the expression of antioxidant genes. It is well documented that CAT enzyme converts H_2_O_2_ into H_2_O and O_2_, and POD enzyme may also play an important role in the catalysis of H_2_O_2_. In addition, increased SOD enzyme activity has been reported to enhance the ability of seedlings to scavenging O_2_−radicals under stress conditions [55]. However, under stress conditions, plants cannot sufficiently eliminate ROS, which can lead to oxidative stress [56]. In this study, the application of GA_3_ significantly reduced the oxidative stress under salt stress, because of the less production of ROS and higher activities of antioxidant enzymes (Figure 4, Figure 5 and Figure 6). Our results also showed that at moderate and severe salinity, GA_3_ application significantly restored antioxidant enzyme activities, as compared to untreated plants. Under severe salinity, the application of GA_3_ increased the activities of antioxidant enzymes and their expression levels, and similar findings have been reported in previously published data [57,58]. Furthermore, GA_3_ application improved the carotenoid (Figure 3), and total phenolic contents (Figure 7), which also protects plants from oxidative damage. In the present study, SOD activity in control plants decreased significantly with increasing the salinity levels. Similar results have previously been reported by Al-Hassan et al. [59] under salt stress.

The restriction in the concentration of Na^+^ ions and maintenance or high level of Ca^2+^ and K^+^ ions are pivotal for plant survival under salt stress [60,61]. Under salinity, higher Ca^2+^ concentration helps plants to maintain their growth [62]. Our results showed that Na^+^ concentration decreased in maize roots and leaves under GA_3_ application. Also, GA_3_ application caused a significant increase in Ca^2+^ and K^+^ ions in different maize parts under salinity stress (Figure 8 and Figure 9). Various studies have shown that the application of GA_3_ significantly reduced the concentration of Na^+^ ions and increased the concentrations of Ca^2+^ and K^+^ ions under salt stress [21,63,64,65]. Interestingly, water spray decreased the Ca^2+^ concentration in maize roots as compared to untreated plants. Perhaps water causes a dilution effect or the priming process itself caused the different responses.

Plants have various defense responses against abiotic stress, including salinity [66], which leads to the production of several secondary metabolites, such as phenolics [67]. In this study, a significant increase in phenolic compounds was reported in GA_3_-treated plants exposed to salt stress (Figure 7). Similar findings were reported in *S. miltiorrhiza* [68] and *Artemisia absinthium* (L.) [69]. Moreover, the role of phenolics in scavenging of ROS in different plant species has also been documented [69,70,71] indicating that higher accumulation of phenolics could be pivotal for plant tolerance against oxidative stress. In this experiment, the application of GA_3_ also significantly increased the total soluble protein under salt stress. These results are consistent with previous reports [72,73], which demonstrated that the application of GA_3_ increased the protein content in maize. The supply of GA_3_ plays an essential role in protein biosynthesis because it can increase the uptake of N from the soil [72]. Accumulation of organic solutes, including soluble protein and phenolics could contribute to osmotic adjustment and stabilization of membranes in plants [58]. It is well-known that organic solutes can enhance plant tolerance against different stresses, including salinity, through maintaining pressure potential and membrane integrity, protection of proteins, and scavenging of free radicals [74,75].

## 5. Conclusions

In summary, salinity stress significantly inhibited the growth and development of maize crop, however, the application of GA_3_ can effectively ameliorate the damage caused by salt stress. Gibberellic acid amendment enhanced the maize growth, chlorophyll content, total soluble protein and K^+^ ion concentration, while reduced the oxidative stress and Na^+^ ion accumulation under salinity. The GA_3_P + GA_3_FS was the most effective treatment for enhancing the growth and development of maize under salt stress. Better stress tolerance and greater growth of maize in this treatment was associated with higher antioxidative defense, maintenance of photosynthetic pigments, higher osmolyte accumulation and better ionic homeostasis in salt-affected soil.

## Data Availability

All the data supporting the findings of this study are included in this article.

## Supplementary Materials

The following are available online at https://www.mdpi.com/article/10.3390/biom11071005/s1, Supplement Table S1: Two-way ANOVA analysis of salt stress, gibberellic acid (GA_3_) application, and their interaction for growth, physiological traits, antioxidant enzymes and ionic contents of maize.
