# Supplementary material for: Exogenously Applied Gibberellic Acid Enhances Growth and Salinity Stress Tolerance of Maize through Modulating the Morpho-Physiological, Biochemical and Molecular Attributes
Source: Biomolecules. 2021 Jul 9;11(7):1005. doi: 10.3390/biom11071005 (PMC8301807; doi:10.3390/biom11071005)
Supplement: Supplementary file 1 [file biomolecules-11-01005-s001.zip › biomolecules-1231920-supplementary.pdf]

Supplementary Information

Exogenously Applied Gibberellic Acid Enhances Growth and Salinity Stress Tolerance of Maize Through Modulating the Morpho-Physiological, Biochemical and Molecular Attributes

Table S1: Two-way ANOVA analysis of salt stress, gibberellic acid (GA_3) application, and their interaction for growth, physiological traits, antioxidant enzymes and ionic contents of maize

	Source of variation		
	Salt treatments	GA_3 application	Salt treatment × GA_3 application
Shoot fresh weight	1099.98**	259.26**	7.45**
Shoot dry weight	1082.45**	277.15**	6.82**
Root fresh weight	935.63**	307.75**	3.80**
Root dry weight	210.01**	74.17**	2.54*
Shoot length	3175.82**	426.51**	15.12**
Root length	878.41**	169.23**	5.94**
Maximum leaf length	274.01**	44.53**	7.29**
Maximum leaf width	313.53**	52.75**	2.58*
Chlorophyll a	128.44**	191.93**	9.73**
Chlorophyll b	135.94**	345.77**	4.50**
Total chlorophyll content	25.79**	205.68**	5.03**
Carotenoid content	47.19**	185.90**	3.03**
Hydrogen peroxide content	604.23**	71.38**	15.23**
Superoxide dismutase	79.51**	60.32**	99.37**
Peroxidase	17.24**	9.68**	50.07**
Catalase	9.31**	24.29**	83.49**
Total soluble protein	151.51**	27.00**	0.84ns
Total phenolic content	161.38**	145.40**	2.84*
Root Na^+	354.98**	44.58**	5.48**
Leaf Na^+	279.67**	94.31**	1.00ns
Leaf K^+	99.15**	36.62**	0.90ns
Root K^+	51.78**	62.06**	1.78ns
Shoot Ca^{2+}	2.68ns	3.79**	0.13ns
Root Ca^{2+}	40.57**	30.81**	2.73**

Data represent F-values. Significant effects are donated as: * - significant at $P \leq 0.05$, ** - significant at $P \leq 0.01$, *** - significant at $P \leq 0.001$, ns – non-significant.
